# Development of Pure Certified Reference Material of Cannabidiol

**DOI:** 10.3390/molecules29050921

**Published:** 2024-02-20

**Authors:** Congcong Yu, Ruihan Long, Feng Cao, Xinying Zhao, Tao Lan, Dunming Xu

**Affiliations:** 1 China National Institute of Standardization, Beijing 100191, China; yucc@cnis.ac.cn; 2 College of Materials and Chemistry & Chemical Engineering, Chengdu University of Technology, Chengdu 610059, China; longruihan@stu.cdut.edu.cn; 3 Department of Materials Chemistry, Huzhou University, Huzhou 313009, China; caofeng@zjhu.edu.cn; 4 College of Bioengineering, Beijing Polytechnic, Beijing 100176, China; zhaoxinying@bpi.edu.cn; 5 Technical Center of Xiamen Customs, Xiamen 361026, China

**Keywords:** cannabidiol, certified reference material, preparative liquid chromatography

## Abstract

Cannabidiol (CBD) is the major functional component in hemp and has a broad range of pharmacological applications, such as analgesic, anti-epileptic, anti-anxiety, etc. Currently, CBD is widely used in pharmaceuticals, cosmetics, and food. To ensure the quality and safety of the products containing CBD, more and more related sample testing is being conducted, and the demand for CBD-certified reference material (CRM) has also sharply increased. However, there is currently a lack of relevant reference materials. In this paper, a simple method for preparing CBD CRM was established based on preparative liquid chromatography using crude hemp extract as a raw material. A qualitative analysis of CBD was performed using techniques such as ultraviolet absorption spectroscopy (UV), infrared spectroscopy (IR), mass spectrometry (MS), nuclear magnetic resonance spectroscopy (NMR), and differential scanning calorimetry (DSC). High-performance liquid chromatography (HPLC) was used for the homogeneity and stability tests, and the data were analyzed using an F-test and a T-test, respectively. Then, eight qualified laboratories were chosen for the determination of a certified value using HPLC. The results show that the CBD CRM had excellent homogeneity and good stability for 18 months. The certified value was 99.57%, with an expanded uncertainty of 0.24% (*p* = 0.95, k = 2). The developed CBD CRM can be used for the detection and quality control of cannabidiol products.

## 1. Introduction

**Figure 1:**
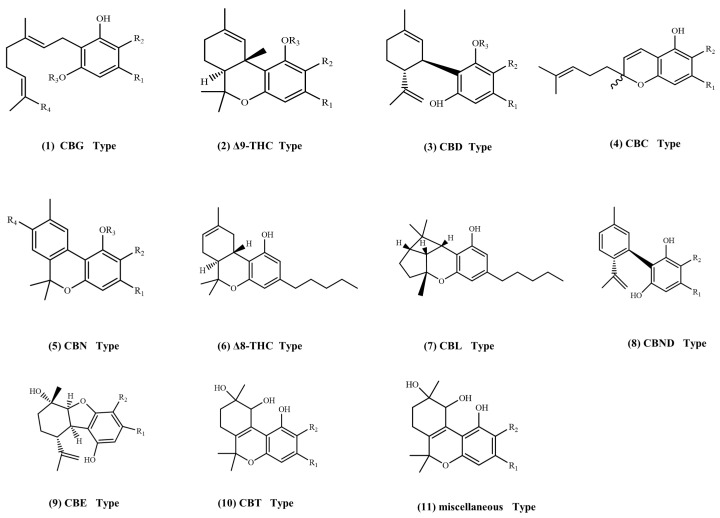
Structural equation of cannabis phenolic compounds.

*Cannabis sativa* L. (*cannabis*) is a controversial plant with a history of cultivation by humans for over a thousand years. Research findings show that over 400 compounds have been extracted and characterized from the *cannabis* plant, among which cannabinoids are the most significant constituents and unique to *cannabis*. They are currently widely studied due to their undeniable medicinal properties [1]. About 144 cannabinoids have been extracted and identified to date, which have been categorized into 11 chemical classes, as displayed in Figure 1: cannabigerol (CBG) type, ^9^-trans-tetrahydrocannabinol (Δ^9^-THC) type, cannabidiol (CBD) type, cannabichromene (CBC) type, cannabinol (CBN) type, ^8^-trans-tetrahydrocannabinol (Δ^8^-THC) type, cannabicyclol (CBL) type, cannabinodiol CBND type, cannabielsoin (CBE) type, cannabitriol (CBT) type, and miscellaneous types [2]. Among these 11 types of cannabinoids, the recent research on cannabidiols has mainly focused on cannabidiol (CBD) and tetrahydrocannabinol (Δ^9^-THC). Δ^9^-THC is a hallucinogenic addictive component and is banned in many countries. But CBD has no hallucinogenic addiction effect and has a broad of pharmacological activities such as anti-inflammatory, analgesic, anti-epileptic, anti-anxiety, anti-spasmodic, etc., and a protective effect on the nervous system and immune system [3,4]. Therefore, many countries have gradually legalized the use of *cannabis*, and cannabidiol-related drugs have been put into clinical trials in Europe, North America, and others. At the same time, various types of foods containing cannabidiol have appeared on the market, including baked foods, beverages, candy, and chocolate [5]. Due to the increasingly wide range of CBD application scenarios, countries have put forward stricter requirements for the quality control of CBD-related products, and their demands for traceable certified reference materials (CRMs) are increasing.

There have been limited investigations into methods for determining the purity of CBD. The availability of precise purity determination methods is crucial for the advancement of CRMs and is significant in establishing measurement traceability, calibrating instruments, and validating experimentation methods. Cannabidiol certified reference material (CBD CRM) is needed for the content detection of cannabidiol, but the supply of CBD CRM is very limited, and CBD CRM is expensive. In addition, the quality of reagents or reference substances currently provided in China cannot be guaranteed, which restricts the rapid and standardized development of cannabidiol-related industries. Therefore, in order to meet the needs of testing and quality control of cannabinoid-related products, it is urgently necessary to independently develop CBD CRM. According to reports in the literature, the current methods for the detection of cannabidiol mainly include high-performance liquid chromatography (HPLC) [6,7], liquid chromatography-tandem mass spectrometry (HPLC-MS/MS) [8,9,10], and gas chromatography with tandem mass spectrometry (GC-MS) [11,12].

**Figure 2:**
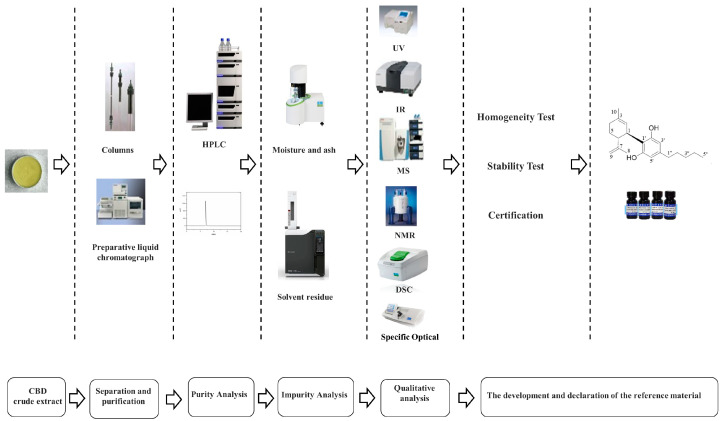
The total development process for the CBD CRM.

In this study, CBD crude extract was utilized as a source material to prepare purified cannabidiol using a series of steps including dissolution extraction, liquid chromatography refinement and purification, solvent removal, and freeze-drying. The resulting purified cannabidiol underwent structural identification using techniques such as ultraviolet absorption spectroscopy (UV), infrared spectroscopy (IR), mass spectrometry (MS), nuclear magnetic resonance (NMR), and differential scanning calorimetry (DSC). Homogeneity tests, stability tests, and the determination of the purity value of CBD were all conducted, resulting in a purity higher than 98% with an expanded uncertainty of less than 1.0%. The total development process for the CBD CRM is shown in Figure 2.

## 2. Results and Discussion

### 2.1. Purity Analysis

#### 2.1.1. Analysis of the CBD Crude Extract

**Figure 3:**
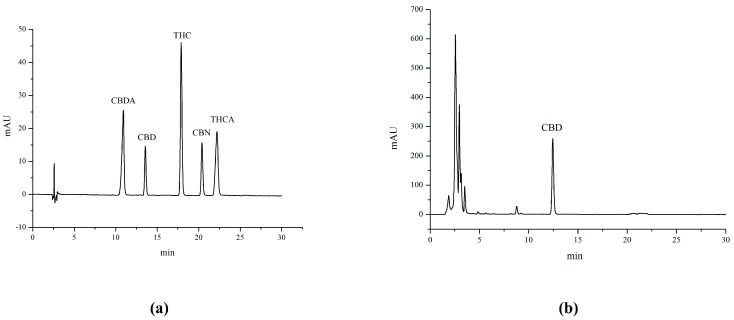
(**a**) Chromatogram of five cannabinoid standards. (**b**) Chromatogram of the CBD crude extract.

The chromatogram of five cannabinoids standards in our developed method is shown in Figure 3a. The chromatogram of the cannabidiol crude extract is displayed in Figure 3b. Figure 3b shows that the primary component of the crude extract was CBD, and it did not contain the prohibited component Δ^9^ tetrahydro-cannabinol (Δ^9^-THC), indicating that the crude extract could be safely used for the preparation of CBD.

#### 2.1.2. Preparation Process for the Purified CBD Product

**Figure 4:**
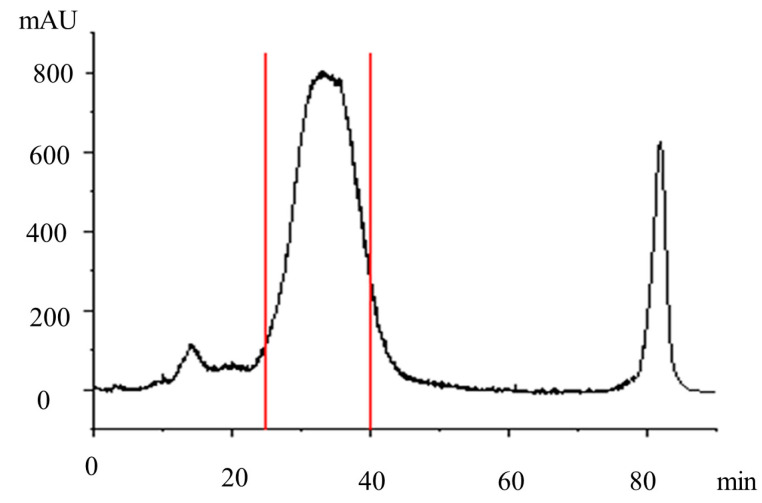
Preparative liquid chromatography of the CBD crude extract. Separation conditions: column: preparation of separation column (C18, HT-ODS-P-10 µm, 20 mm × 250 mm, PAT-201207-1). Mobile phase: 85% methanol +15% water; flow rate: 1.5 mL/min; column temperature: 30 °C; detection wavelength: 220 nm; injected volume: 0.5 mL; CBD concentration: 500 mg/mL.

The purified cannabidiol fraction was collected and obtained using the preparative liquid chromatography system. Then, the collected sample was subjected to vacuum concentration for the purified cannabidiol liquid. The prepared chromatogram is shown in Figure 4. Then, the purified cannabidiol liquid was freeze-dried to obtain the purified CBD product.

#### 2.1.3. Purity Analysis of the Purified CBD Product

**Figure 5:**
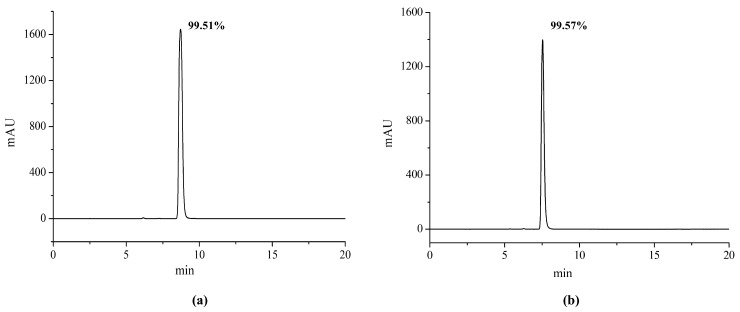
(**a**) Chromatogram of the purified cannabidiol product based on the column from Elite (Su persil-ODS2, 250 mm × 4.6 mm, 5 μm). (**b**) Chromatogram of the purified cannabidiol product based on the column from Agilent (ZORBAX SB-C18, 250 mm × 4.6 mm, 5 μm). Separation conditions: column temperature: 35 °C; injection volume: 10 μL; mobile phase: 0.1% formic acid in water: 0.1% formic acid in acetonitrile = 20:80 (*v*/*v*); flow rate: 1 mL/min; detection wavelength: 220 nm.

**Figure 6:**
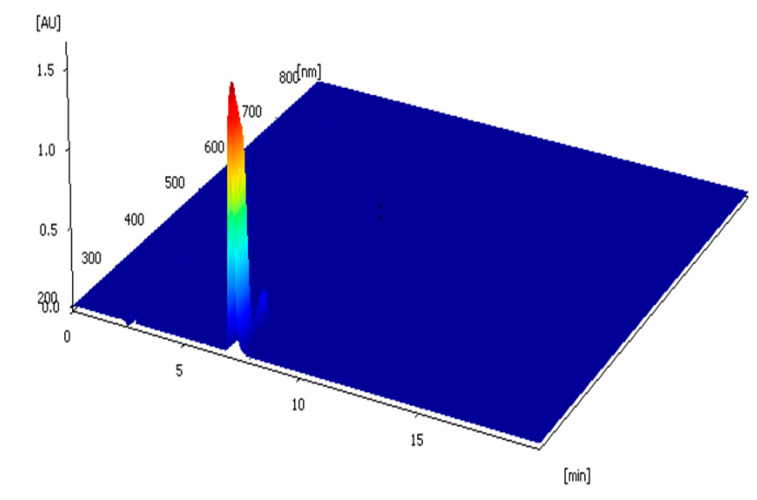
Three-dimensional spectral scan of the purified CBD product. Separation conditions: column: ZOBRAX SB-C18, (250 mm × 4.6 mm, 5 μm); column temperature: 35 °C; injection volume: 10 μL; mobile phase: 0.1% formic acid in water: 0.1% formic acid in acetonitrile = 20:80 (*v*/*v*); flow rate: 1 mL/min; detection wavelength: 220 nm.

The CBD crude extract underwent the preparative liquid chromatography system, and about 5.3 g of purified cannabidiol product was obtained. The chromatograms obtained under the isocratic elution from the Agilent and Elite columns are shown in Figure 5. They revealed the absence of significant impurity peaks in both chromatograms. The purity values of CBD were 99.51% and 99.57%, respectively, based on the quantitative analysis of the chromatographic peak with area normalization, which met the standard requirements. The 3D full-spectrum scanning in the range of 200–800 nm for CBD based on the ZORBAX SB-C18 column is shown in Figure 6, which also showed no impurity peaks.

**Figure 7:**
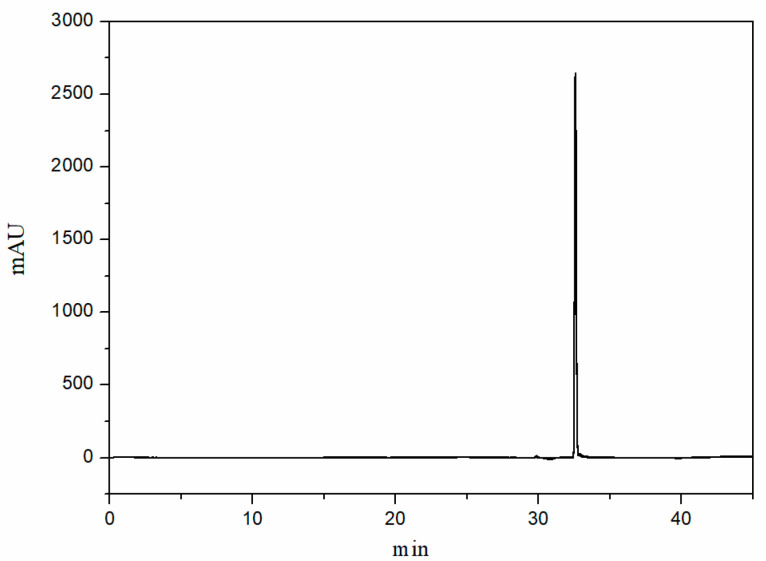
Chromatogram of the purified cannabidiol product based on the column from Agilent (ZORBAX SB-C18, 250 mm × 4.6 mm, 5 μm). Separation conditions: column temperature: 35 °C; injection volume: 10 μL; mobile phase: 0.1% formic acid in water: 0.1% formic acid in acetonitrile = 20:80 (*v*/*v*); flow rate: 1 mL/min; detection wavelength: 220 nm. Gradient program: 0–30 min, 10%B–90%B; 30–35 min, 90%B–90%B; 35.1 min, 10%B; 35.1–45 min, 10%B–10%B.

In addition, the purity analysis of CBD under gradient elution was also investigated using the ZORBAX SB-C18 column. In the chromatogram shown in Figure 7, it can be seen that there were no obvious impurity peaks, and the purity value of CBD was 99.78% based on the quantitative analysis of the chromatographic peak with area normalization, which could meet the standard requirements.

#### 2.1.4. Impurity Analysis of the Purified CBD Product

**Figure 8:**
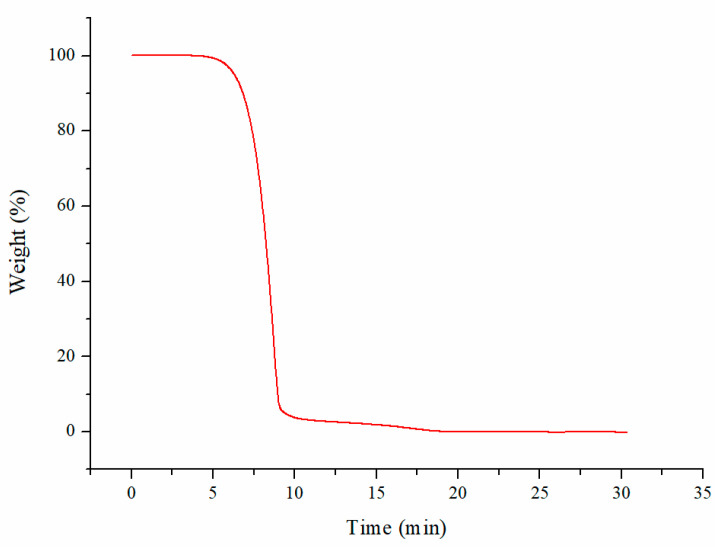
TG curve of cannabidiol determination.

The thermogravimetric analysis method is simple and fast, and the moisture and ash content can be simultaneously determined in a single sample with only a small amount of the sample. Therefore, we chose thermogravimetric analysis for the determination of moisture and ash in this work as our preparation quantity was very small. The thermogravimetric (TG) curve for CBD is shown in Figure 8. Using the curve, the weight loss at 105 °C could be calculated as the moisture content value and the residual amount at the end of the combustion could be calculated as the ash content value. After the measurement, the content of moisture and ash were both < 0.1% with a relative standard deviation (RSD) <0.01%. The data obtained from three parallel measurements of the CBD samples are presented in Table 1. 

**Figure 9:**
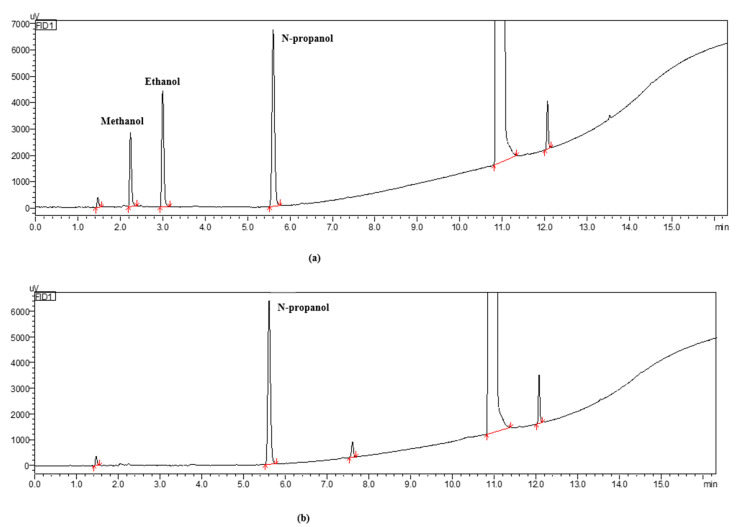
(**a**) Gas chromatograms of the methanol standard, ethanol standard, and n-propanol standard. (**b**) Gas chromatograms of the purified cannabidiol product.

The contents of methanol and ethanol used in the preparation were detected based on the gas chromatogram, and the chromatograms are shown in Figure 9. In the chromatogram of the CBD sample in Figure 9b, it can be seen that methanol and ethanol solvents were not detected. Since the headspace sampling method is suitable for the determination of residual volatile organic solvents, the quantification of difficult volatile organic solvents was inaccurate. Moreover, the detection of other types of solvent residues was not taken into account in this method, and thus, there were certain errors in the determination of the solvent residues.

### 2.2. Qualitative Analysis

UV, IR, MS, NMR, and DSC were used for the structure identification of CBD. The related characterization materials are shown in Supplementary Figures S1–S6. As shown in Figure S1, the UV absorption spectra revealed that CBD had two maximum absorption wavelengths, λ_1_ = 210 nm and λ_2_ = 273 nm, which were consistent with those reported in the literature [13].

Figure S2 shows the mass spectrum of CBD. The results showed that the *m/z* of the substance in the negative ion mode was 313.2164, and 627.4401 was speculated to be the hydrogenation peak of the dimer C_42_H_59_O_4_. The data for the two peaks was basically the same as CBD; therefore, it was speculated that the compound was CBD with the molecular formula of C_21_H_30_O_2_.

Figure S3 shows the IR spectrum of CBD. The characteristic wavenumbers at 3521 cm^−1^ and 3410 cm^−1^ were consistent with the phenolic hydroxyl groups on the benzene ring. The characteristic wavenumber at 3074 cm^−1^ was demonstrated to be the stretching vibration of hydrocarbon bonds on the benzene ring. The characteristic wavenumber at 3031 cm^−1^ corresponded to the C-H stretching vibration of -CH=CH-. The characteristic wavenumber at 2966 cm^−1^ corresponded to the C-H stretching vibrations of CH_3_, and the characteristic wavenumber at 2926 cm^−1^ corresponded to the =CH. The characteristic wavenumbers at 1623 cm^−1^, 1582 cm^−1^, 1514 cm^−1^, and 1444 cm^−1^ proved to be the stretching vibration peak of the benzene ring of -C=C-. The characteristic wavenumber at 1375 cm^−1^ corresponded to the C-H deformation vibration of CH_3_. The results were highly consistent with the description of CBD reported in the literature and the standard spectra of CBD, indicating that the compound was CBD.

**Figure 10:**
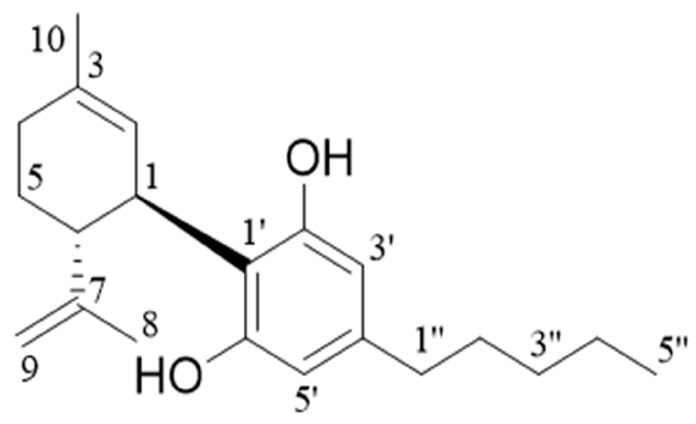
The structural equation of CBD CRM.

Figures S4 and S5 show the ^1^HNMR and ^13^CNMR spectra of CBD, and the analysis of spectra is shown in Table 2. According to the structural formula of CBD shown in Figure 10, the results of CBD in our work were consistent with the data reported in the literature [14].

Differential scanning calorimetry (DSC) was used to measure the melting temperature of the CBD CRM. The DSC curve of the CBD CRM is shown in Figure S6. The results showed that the melting temperature (*T_m_*) of the CBD CRM was 66.1~66.7 °C, which agreed with data available in the literature [15].

A fully automatic polarimeter was used to measure the specific rotation of CBD, and the results showed that the specific rotation value of CBD was −123.4° after the calculation. The CBD had a levorotation configuration, which is consistent with the literature [16].

### 2.3. Homogeneity Test

The result of the homogeneity test is presented in Table 3. An F-test [17,18] was used to determine whether the uniformity data of the CBD CRM conformed to a normal distribution according to the ISO Guide 35:2017 [19,20]. The analysis of variance (ANOVA) for the homogeneity study is provided in Table 4. Based on the data in Table 4, the mean square value between the group (*S*_1_^2^) and the mean square value within the group (*S*_2_^2^) was calculated. As data shown in Table 3, the corresponding *F* value was calculated using Equation (1)

*F* = *S*_1_^2^/*S*_2_^2^ (1)

The result shows that *F* = 1.9408 < *F*_0.05_ (11, 24), indicating the CBD CRM was uniform and the homogeneity did not differ significantly. Then, the uncertainty in uniformity (*u_bb_*) was calculated using Equation (2), where *n* is the number of measurements

(2) $$u_{bb}=\sqrt{\frac{{(S}_{1}^{2}-S_{2}^{2})}{n}}$$

Therefore, the uncertainty in the homogeneity measurement *u_bb_* was 0.04%.

### 2.4. Stability Test

#### 2.4.1. Short-Term Stability

The short-term stability test for the CBD CRM was carried out at 4 °C, 25 °C, and 60 °C for 7 days. The results are displayed in Table 5. The purity was determined on days 1, 3, and 7, and the average purity values were calculated, respectively. The results indicated that CBD could remain stable for a short period at three temperatures, which provided a reference for the transportation conditions.

#### 2.4.2. Long-Term Stability

**Figure 11:**
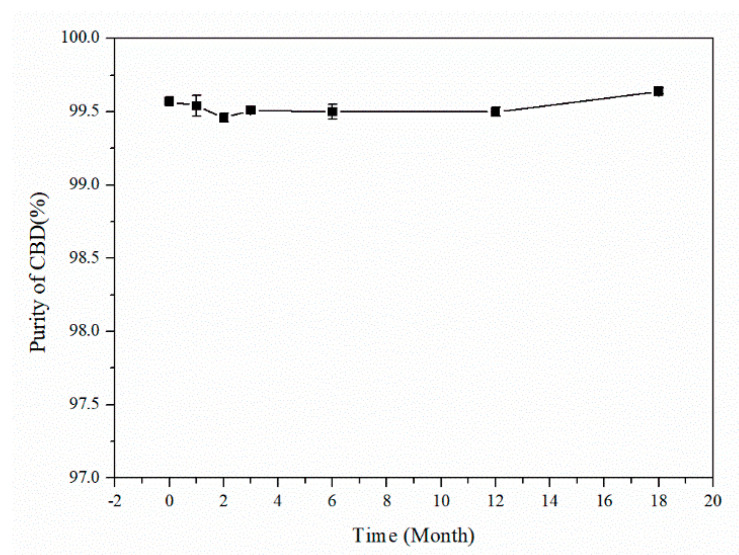
The trend in the purity of the CBD CRM.

In accordance with the requirements of the ISO Guide 35: 2017 [19,20], a T-test [17,18] was used for the long-term stability evaluation of the CBD CRM and the calculation of uncertainty in stability (*u_s_*) over 18 months. The determined results in Table 6 and the trend in the purity in Figure 11 show that the obtained average values throughout the assessment period exhibited no decreasing trend over time. The evaluation process was realized using the following Equations (3)–(5)

(3) $$b_{1}=\frac{\sum_{i=1}^{n}\left(X_{i}-\bar{X})(Y_{i}-\bar{Y}\right)}{\sum_{i=1}^{n}{\left(X_{i}-\bar{X}\right)}^{2}}$$

(4) $$s^{2}=\frac{\sum_{i=1}^{n}{\left(Y_{i}-b_{0}-b_{1}X_{i}\right)}^{2}}{n-2}$$

(5) $$s\left(b_{1}\right)=\frac{s}{\sqrt{\sum_{i=1}^{n}{\left(X_{i}-\bar{X}\right)}^{2}}}$$

where *b*_1_ is the slope and *s(b*_1_*)* is the uncertainty in the slope *b*_1_. The critical t-value at a 95% confidence level is *t*_0.95,*n*−2_ = 2.57. The calculation results showed that $\left|b_{1}\right|<t_{0.95,\;n-2}\times s\left(b_{1}\right)$, indicating that the data were judged to be effective and there was no significant change in the stability under long-term storage. Then, the uncertainty in stability (*u_s_*) was calculated according to Equation (6), where *X* stands for the time of long-term storage. Based on the calculation, the uncertainty in the CBD CRM preservation for 18 months was 0.09%.

(6) $$u_{s}=s\left(b_{1}\right)\cdot X$$

#### 2.4.3. Certification of the CBD CRM

The certificated values for the purity of CBD CRM using HPLC from eight laboratories are shown in Table 7. The results show that the average values ranged from 99.49% to 99.83% with an RSD< 0.05%. According to the requirements of the ISO Guide 35:2017, all the data were valid based on the calculation using the Grubbs method. Then, the average values from eight laboratories were statistically evaluated using ANOVA. The results are shown in Table 8, and the uncertainty in the certification of the CBD CRM was calculated.

For the ANOVA using the data in Table 8, the between-laboratory standard deviation was named *S*_L_, and the repeatability standard deviation was named *S*_r_, and they were calculated using Equations (7) and (8), respectively. The uncertainty in the certification (*u_char_*) was calculated using Equation (9). The certificated value of CBD CRM was 99.68%, and the *u_char_* value was 0.05%.

(7) $$S_{L}=\sqrt{\frac{{MS}_{between}-{MS}_{within}}{n_{0}}}$$

(8) $$S_{r}=\sqrt{{MS}_{within}}$$

(9) $$u_{char}=u\left(\overset{̿}{X}\right)=\sqrt{\left(\frac{S_{L}^{2}}{p}+\frac{S_{r}^{2}}{n\cdot p}\right)}$$

where

*MS_between_*—The between-group mean squares;
*MS_within_*—The within-group mean squares;
*n*—The number of the laboratory;
*p*—The number of the determination in each laboratory;
$\overset{̿}{X}$—The average value of the total.

Then, the certificated value of CBD was calculated using Equation (10), where *I* represents the mass fraction of CBD and *I_organic_*, *I_moisture_*, *I_ash_*, and *I_solvent_* represents the content of organic impurity, moisture, ash, and residual solvent, respectively. The certificated value of CBD was found to be 99.57% using the calculation:

(10) $$I=\left(100\%-I_{organic}\right)\times \left(100\%-I_{moisture}-I_{ash}-I_{solvent}\right)\times 100\%$$

### 2.5. Uncertainty Assessment

According to the ISO Guide 35:2017, the certificated values of the reference material consisted of the standard value and the expended uncertainty (*U_CRM_*). The *U_CRM_* can be estimated by multiplying the combined uncertainty (*u_CRM_*) and the coverage factor (*k* = 2) at the confidence level of 95%. The *u_CRM_* is composed of the certification (*u_char_*), the uncertainty in the homogeneity measurement (*u_bb_*), the uncertainty in the stability measurement (*u_s_*), the uncertainty in the moisture measurement (*u_m_*), the uncertainty in ash measurement (*u_a_*), and the uncertainty in solvent residue measurements (*u_sr_*). The *u_CRM_* and *U_CRM_* values were calculated using Equations (11) and (12).

(11) $$u_{CRM}=\sqrt{\left(u_{char}^{2}+u_{bb}^{2}+u_{s}^{2}+u_{m}^{2}+u_{a}^{2}+u_{sr}^{2}\right)}$$

(12) $$U_{CRM}=k\cdot u_{CRM}$$

As the moisture content for CBD was 0.035%, which was lower than 0.1%, the *u_m_* could be ignored. Similarly, the *u_a_* was also ignored as the ash content was 0.072%. No residual solvent was detected, and the *u_sr_* was also ignored. Thus, the *u_CRM_* value of CBD CRM was 0.12%, and the *U_CRM_* value was 0.24%.

## 3. Materials and Methods

### 3.1. Apparatus and Reagents

Chemicals: The 60% cannabidiol crude extract was produced by Tianzhilu Biotechnology Co., Ltd. (Xi’an, China). The HPLC-grade methanol and acetonitrile were from Fisher Scientific (Hampton, NH, USA). The formic acid was from Sinopharm Chemical Reagent Beijing Co., Ltd. (Beijing, China).

Instruments: Rotary evaporator RE-2000 (Shanghai Yarong Biochemical Instrument Factory, Shanghai, China); LC-8A semi-preparative high-performance liquid chromatography system (Shimadzu, Tokyo, Japan); freeze-drying machine (Labconco, Kansas City, MI, USA); iChrom W5100 high-performance liquid chromatograph (Dalian Elite Analytical Instrument Co., Ltd., Dalian, China); TGA 8000 thermo-gravimetric analyzer (PerkinElmer Co., Ltd., Waltham, MA, USA); Nexis GC-2030 gas chromatograph (Shimadzu, Tokyo, Japan); G10S UV-Vis spectrophotometer (Thermo Fisher Scientific, Waltham, MA, USA); IR Prestige-21 mid-infrared spectrometer (Shimadzu, Tokyo, Japan); high-performance liquid chromatograph in series with a TSQ Quantum Access MAX triple quadrupole mass spectrometer (Thermo Fisher Scientific, Waltham, MA, USA); DSC8000 differential scanning calorimeter (PerkinElmer Co., Ltd., Waltham MA, USA); BRUKER 400 MHz nuclear magnetic resonance spectrometer (Bruker, Billerica, MA, USA); and Autopol IV Automatic polarimeter (Rudolph Research Analytical, Wilmington, DE, USA).

### 3.2. Extraction and Purification of CBD

#### 3.2.1. Purity Analysis of the CBD Crude Extract

The developed method [21] for the detection of five cannabinoids using HPLC by our team was used to analyze the composition of the 60% cannabidiol crude extract with a Supersil-ODS2 column (4.6 mm × 250 mm, 5 μm). The column temperature was maintained at 35 °C, the injection volume was 10 μL, and 0.1% formic acid in water (A) and 0.1% formic acid in acetonitrile (B) were used as the mobile phase at a flow rate of 1 mL/min with a detection wavelength of 220 nm. The system was run with the following gradient program: 0–20 min, 70%B–90%B; 20–22 min, 90%B; 22–24 min, 90%B–70%B; and 24–30 min, 70%B.

#### 3.2.2. Isolation and Purification of CBD

A high-pressure purification system was used for the further purification of 15.3 g of the 60% cannabidiol crude extract with the preparation of a C18 column (HT-ODS-P-10 µm, 20 mm × 250 mm, PAT-201207-1). Elution was carried out with the mobile phase combined with 85% methanol and 15% water at a flow rate of 1.5 mL/min. The column temperature was held at 30 °C, and the detection wavelength was 220 nm. CBD at a concentration of 500 mg/mL was injected with a volume of 0.5 mL, and then the cannabidiol fraction was collected. Throughout the concentration under reduced pressure, the purified cannabidiol liquid was obtained. Later the solvent was removed using rotary evaporation, and the sample was freeze-dried to obtain the highly purified cannabidiol product.

### 3.3. Analysis of the Purified CBD Product Purity

HPLC was used on the iChrom W5100 to analyze the purity of the obtained cannabidiol, and the different chromatographic conditions were optimized including different columns and different wavelengths. For the different columns, the temperature of the column was maintained at 35 °C with an injection volume of 10 μL. The detection wavelength was 220 nm. Then, the ZORBAX SB-C18 column (250 mm × 4.6 mm, 5 μm) from Agilent and the Supersil ODS2 column (250 mm × 4.6 mm, 5 μm) from Dalian Elite were compared for the detection of CBD with the mobile phase consisting of 80% acetonitrile and 20% water (*v*/*v*) at a flow rate of 1.0 mL/min. Additionally, for the different wavelengths, the CBD sample was detected at continuous scanning wavelengths from 200 nm to 800 nm under the previously optimized conditions.

The thermos-gravimetric analysis of CBD was carried out on the TGA 8000 for the measurement of moisture and ash content under an air atmosphere. The temperature ranged from 40 °C to 800 °C with a heating rate of 30 °C/min, and then the temperature was held at 800 °C for 5 min. The measurement of CBD was performed in three parallel experiments, and the average results were separately calculated for the contents of moisture and ash.

The solvent residue in CBD was determined using gas chromatography on the Nexis GC-2030. The determination followed the method for solvent residue determination described in the Chinese Pharmacopoeia 2020 edition: Part IV General Notices 0861. The methods of headspace sampling and internal standard single-point control were used in this measurement, with n-propanol as the internal standard. The SHIMADZU Rtx-624 (30.0 m × 0.32 mm × 1.80 μm) column was used with the forward sample port temperature at 200 °C. The detector temperature was 220 °C, and the injection volume was 1 mL. The split ratio was 10:1, and the column flow rate was 2.0 mL/min. The temperature was first held at 40 °C for 5 min, increased to 180 °C at a rate of 15 °C/min, and then was held for 2 min. Then, the methanol and ethanol solvent residues in the CBD sample were detected. The measurement was performed in three parallel experiments, and the average results for the contents of methanol and ethanol were calculated, respectively.

### 3.4. Qualitative Analysis

According to the ISO Guide35:2017 [19], the qualitative analysis of CBD was realized using UV, IR, MS, and NMR for structure identification. The scanning range of UV was 190~800 nm, and the scanning range of IR was 400~4000 cm^−1^. The negative ion full scan model was carried out with the parent ion range of 100~1000 with MS. CBD was dissolved in CDCl_3_ with tetramethylsilane as the internal standard for the NMR test.

Meanwhile, the differential scanning calorimetry (DSC) curve of CBD was also obtained for the melting temperature on the DSC 8000 from PerkinElmer. The CBD powder was heated from 60 °C to 70 °C with a heating rate of 0.5 °C/min. Then, the melting temperature was determined as the onset temperature and the melting enthalpy as the peak area. In addition, an optical rotation experiment was also carried out for the configuration information on the automatic polarimeter. The concentration of CBD was 0.01 g/mL dissolved in methanol, and the test was carried out in a calibrated polarimeter at 589 nm at 20 °C. Then, the specific rotation was calculated. All the results of CBD obtained with the UV, IR, MS, NMR, DSC, and optical rotation experiments were compared with values reported in the literature.

### 3.5. Homogeneity Test

An electronic microbalance (0.0001 g) was used to pack the purified CBD products in a cleanroom. Then, 10 mg CBD was divided into brown sample bottles numbered from 1 to 100. The packaged sample bottles were then placed in a sample box and stored in a refrigerator at 4 °C.

The homogeneity test for CBD CRM was conducted according to the requirements of ISO Guide 35: 2017 [19]. Twelve bottles of samples were randomly selected from the 100 packaging units for the homogeneity testing with three replicates. The samples at the concentration of 1.0 mg/mL dissolved with the mobile phase were determined using HPLC, and the purity value of the samples was read by area percentage. Finally, the uniformity in the CBD was judged based on the ANOVA for the test results.

### 3.6. Stability Test

According to the requirements of ISO Guide 35:2017 [19], the stability test for CBD was conducted. The short-term stability and long-term stability of CBD were studied in this work.

#### 3.6.1. Short-Term Stability Test

Three randomly selected bottles were separately placed at 4 °C, 25 °C, and 60 °C for the short-term stability test of CBD. The study lasted for 7 days, with purity inspections performed on days 1, 3, and 7. The average purity value of each sample was determined from three measurements.

#### 3.6.2. Long-Term Stability Test

Fourteen bottles of CBD divided into seven groups were stored at 4 °C, and the long-term stability test of CBD was checked using HPLC at 0, 1, 2, 3, 6, 12, and 18 months, respectively. Each sample was tested in triplicate. The average purity value of samples in the same group was considered as the final purity value.

### 3.7. Certification

The purity value of CBD was certificated following the requirements of ISO Guide 35:2017 [19], and eight laboratories with China National Accreditation on Service for Conformity Assessment (CNAS) or China Inspection Body and Laboratory Mandatory Approval (CMA) qualifications in this field were selected for the collaborative testing of CBD. Sixteen bottles of CBD from the packaged samples were distributed to the eight laboratories, and each laboratory received two bottles. The CBD from each of the two bottles was quantitatively analyzed at the concentration of 1 mg/mL for three parallel measurements using HPLC. Finally, the purity of the CBD CRM was calculated using area normalization. Then, the results of the certification from eight laboratories were analyzed.

## 4. Conclusions

In order to solve the problem that domestically available CBD CRM is not available, our work developed a simple purification method for its preparation successfully. The structure identification of CBD using UV, IR, MS, and NMR met the results reported in the literature, as well as the melting temperature and the specific rotation. Based on the method for the purity determination using HPLC, the CBD CRM was demonstrated to have good homogeneity and kept its stability for 18 months. Meanwhile, collaborative testing was carried out for the certification of the purity values. The results showed that the certification value of the CBD CRM was 99.57% with an expanded uncertainty of 0.24% (*p* = 0.95%, k = 2). According to the results of this work, the CBD CRM could keep good stability under suitable storage conditions. To achieve the best effect, it is recommended to store the CBD CRM at 4 °C in a dark refrigerator for long-term storage. Cold chain transportation is necessary if the CBD CRM needs to be transferred to ensure the sample is in a better state. The developed CBD CRM provides technical support and value traceability assurance for the analysis of cannabidiol products and related fields, ensuring the quality control of products on a global scale. Moreover, it contributes to standardizing and guiding the rapid development of the global cannabinoid industry, enabling the unique medicinal value of CBD to serve human health better. As the preparation amount was not large in this study, if the industry needs more reference materials, this method needs to be further amplified and optimized. Next, we will focus on developing some other certification reference materials related to cannabinoids for the development and standardization of the domestic cannabinoid industry.

## Figures and Tables

**Table 1:** Moisture and ash determination results of cannabidiol.

Number	1	2	3	Mean (%)	RSD (%)
Moisture content (%)	0.036	0.037	0.032	0.035	0.003
Ash content (%)	0.077	0.072	0.068	0.072	0.005

**Table 2:** Nuclear magnetic resonance hydrogen spectrum data of cannabidiol.

Number	^1^H-NMR δ/ppm	Value in the Literature [14] δ/ppm	Assignment	^13^C-NMR δ/ppm	Value in the Literature [14] δ/ppm
1	3.83	3.86	^1^H, br d, J = 8.4 Hz	37.3	37.0
2	5.57	5.55	^1^H, s	124.1	124.3
3	-	-	-	140.0	139.9
4	2.22, 2.11	2.20, 2.10	^1^H, m; 1H, m	31.5	31.5
5	1.81	1.84	^2^H, m	28.4	28.4
6	2.40	2.40	^1^H, m	46.1	46.2
7	-	4.40	^2^H, m	149.9	149.9
8	1.66	1.66	^3^H, s	20.5	20.3
9	4.67, 4.66, 4.56	4.40	^2^H, m	110.8	110.8
10	1.79	1.79	^3^H, s	23.7	23.4
1′	-	-	-	113.7	113.8
2′	5.97	-	^1^H, br s, 2′-OH	156.1	156.0
3′	6.28	6.26	^1^H, br s	108.0	108.3
4′	-	-	-	143.0	142.9
5′	6.16	6.16	^1^H, br s	109.8	108.3
6′	4.60	-	^1^H, br s, 6′-OH	153.9	153.9
1′′	2.43	2.42	^2^H, t, J = 7.7 Hz	35.5	35.5
2′′	1.56	1.57	^2^H, m	30.4	30.4
3′′	1.30	1.30	^2^H, m	30.6	30.7
4′′	1.31	1.31	^2^H,	22.5	22.5
5′′	0.88	0.89	^3^H, t, J = 6.0 Hz	14.0	14.1

**Table 3:** Homogeneity test results of the CBD CRM.

Number	1	2	3	4	5	6	7	8	9	10	11	12
1 (%)	99.75	99.66	99.65	99.74	99.78	99.79	99.73	99.77	99.76	99.67	99.65	99.72
2 (%)	99.77	99.71	99.73	99.67	99.65	99.76	99.75	99.83	99.78	99.58	99.67	99.65
3 (%)	99.70	99.79	99.75	99.69	99.66	99.78	99.81	99.68	99.67	99.69	99.68	99.69
Mean (%)	99.74	99.72	99.71	99.70	99.70	99.78	99.76	99.76	99.74	99.65	99.67	99.69
Overall Mean (%)	99.72											

**Table 4:** ANOVA for the uniformity study of the CBD CRM.

Variation Source	Degrees of Freedom	Values
Mean square between groups	9	*S*_1_^2^ = 0.005875
Mean square within groups	20	*S*_2_^2^ = 0.003027
*F*_0.05_ (11,24)		2.22
*F*		*F* = *S*_1_^2^/*S*_2_^2^ = 1.9408

**Table 5:** Short-term stability test results of the CBD CRM.

	Time (Day)	Values (%)			Means (%)	RSD (%)
		1	2	3		
4 °C	1	99.68	99.66	99.65	99.66	0.02
	3	99.64	99.67	99.67	99.66	0.02
	7	99.65	99.68	99.67	99.67	0.02
25 °C	1	99.66	99.63	99.62	99.64	0.02
	3	99.67	99.53	99.61	99.60	0.07
	7	99.56	99.67	99.65	99.63	0.06
60 °C	1	99.71	99.68	99.67	99.69	0.02
	3	99.66	99.67	99.69	99.67	0.03
	7	99.66	99.69	99.67	99.67	0.02

**Table 6:** Long-term stability test results for CBD CRM.

Time (Month)	Values (%)	Means (%)	RSD (%)	Uncertainty (%)
0	99.54 99.60 99.57	99.57	0.03	0.09
1	99.51 99.62 99.49	99.54	0.07	
2	99.47 99.44 99.49	99.46	0.03	
3	99.53 99.49 99.51	99.51	0.02	
6	99.45 99.55 99.50	99.50	0.05	
12	99.53 99.48 99.49	99.50	0.03	
18	99.62 99.65 99.66	99.64	0.02	

**Table 7:** Certification results of cannabidiol.

Number	Measured Value (%)						Mean (%)	SD (%)	RSD (%)
	1–1	1–2	2–1	2–2	3–1	3–2			
1	99.51	99.49	99.48	99.48	99.50	99.50	99.49	0.01	0.01
2	99.72	99.72	99.72	99.72	99.72	99.72	99.72	0.00	0.00
3	99.55	99.52	99.52	99.52	99.52	99.53	99.53	0.02	0.02
4	99.62	99.62	99.62	99.60	99.59	99.60	99.61	0.01	0.01
5	99.79	99.90	99.81	99.82	99.82	99.83	99.83	0.04	0.04
6	99.72	99.72	99.72	99.72	99.72	99.72	99.72	0.00	0.00
7	99.80	99.81	99.81	99.78	99.78	99.78	99.79	0.01	0.01
8	99.71	99.71	99.73	99.74	99.72	99.72	99.72	0.01	0.01

**Table 8:** Statistical analysis table of the determined value results.

Number of the Laboratory	1	2	3	4	5	6	7	8
Mean (%)	99.49	99.72	99.53	99.61	99.83	99.72	99.79	99.72
RSD (%)	0.01	0.00	0.01	0.01	0.04	0.00	0.01	0.01
$\overset{̿}{X}$ (%)	99.68							
*S* (%)	0.12							
*MS_between_*	0.088521							
*MS_within_*	0.0063875							
*S*_r_ (%)	0.08							
*S*_L_ (%)	0.12							
*u* (%)	0.05							

## Data Availability

The data are contained within this article and the Supplementary Materials.

## Supplementary Materials

The following supporting information can be downloaded at: https://www.mdpi.com/article/10.3390/molecules29050921/s1, Figure S1. The UV spectra of CBD CRM; Figure S2. The mass spectrum of CBD CRM in the negative ion mode; Figure S3. The IR spectra of CBD CRM; Figure S4. The ^1^HNMR spectra of CBD CRM; Figure S5. The ^13^CNMR spectra of CBD CRM; Figure S6. The DSC curve of CBD CRM.
