# Supplementary material for: Development of Pure Certified Reference Material of Cannabidiol
Source: Molecules. 2024 Feb 20;29(5):921. doi: 10.3390/molecules29050921 (PMC10935364; doi:10.3390/molecules29050921)
Supplement: Supplementary file 1 [file molecules-29-00921-s001.zip › molecules-2726966-supplementary.pdf]

Supplementary

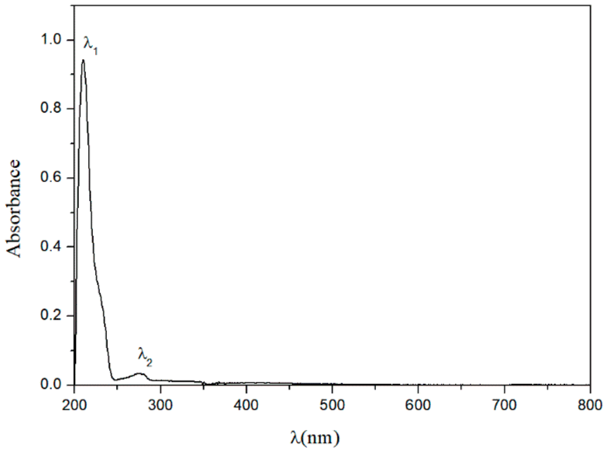

Figure S1. The UV spectra of CBD CRM

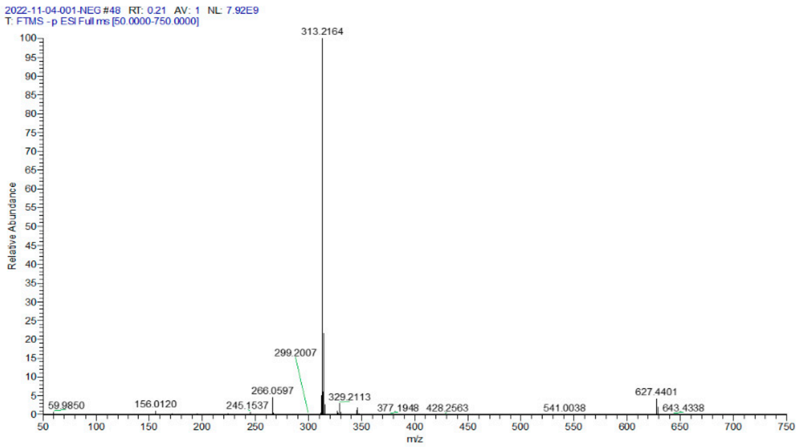

Figure S2. The mass spectrum of CBD CRM in negative ion mode

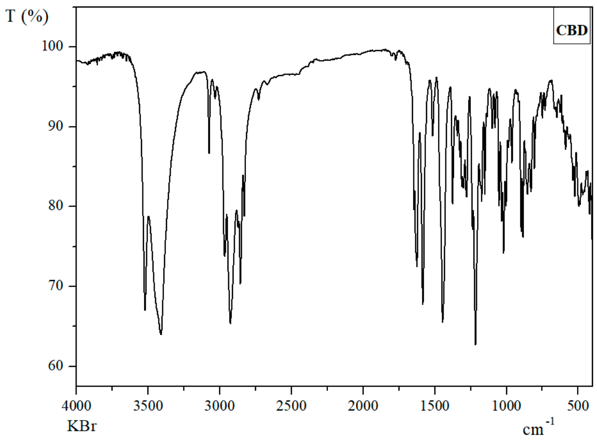

Figure S3. The IR spectra of CBD CRM

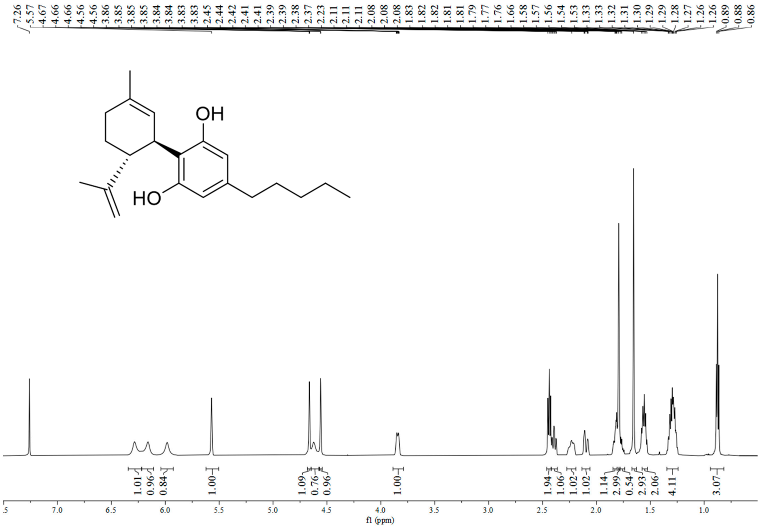

Figure S4. The ^{1}HNMR spectra of CBD CRM

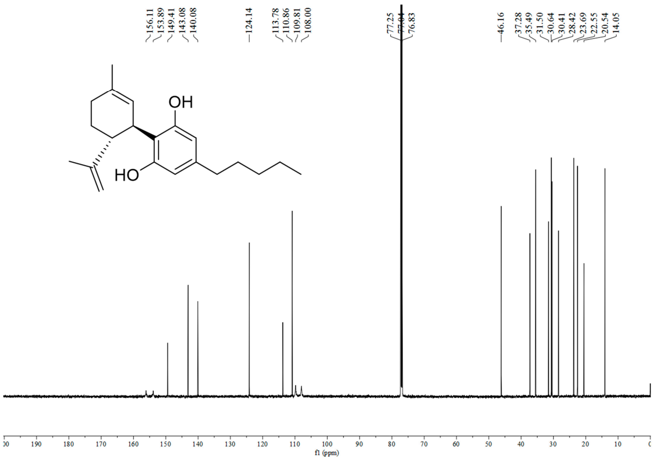

Figure S5. The ^{13}CNMR spectra of CBD CRM

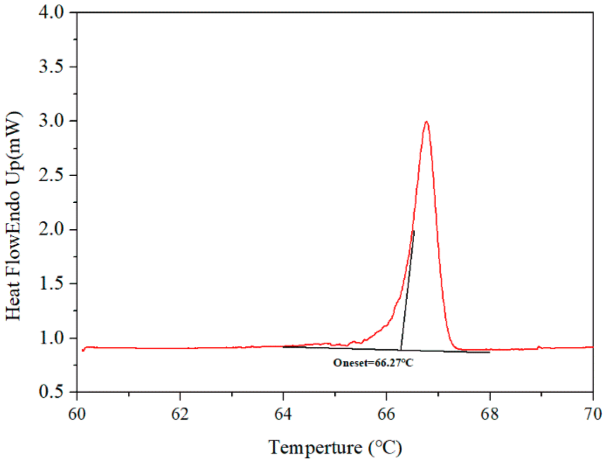

Figure S6. The DSC curve of the CBD CRM
